# Real-Time Fluorescence Monitoring System for Optimal Light Dosage in Cancer Photoimmunotherapy

**DOI:** 10.3390/ph17091246

**Published:** 2024-09-22

**Authors:** Hideki Tanaka, Yoshikatsu Koga, Mayumi Sugahara, Hirobumi Fuchigami, Akihiro Ishikawa, Toru Yamaguchi, Akiko Banba, Takeshi Shinozaki, Kazuto Matsuura, Ryuichi Hayashi, Shingo Sakashita, Masahiro Yasunaga, Tomonori Yano

**Affiliations:** 1 Department of Head and Neck Surgery, National Cancer Center Hospital East, Kashiwa 277-8577, Japan; hidetana@east.ncc.go.jp (H.T.); kmatsuur@east.ncc.go.jp (K.M.);; 2 Department of Otorhinolaryngology, Head and Neck Surgery, Tokyo Medical University, Shinjuku 160-0022, Japan; 3 Department of Strategic Programs, Exploratory Oncology Research & Clinical Trial Center, National Cancer Center, Kashiwa 277-8577, Japan; ykoga@east.ncc.go.jp; 4 Department of Gastroenterology and Endoscopy, National Cancer Center Hospital East, Kashiwa 277-8577, Japan; 5 Division of Developmental Therapeutics, Exploratory Oncology Research & Clinical Trial Center, National Cancer Center, Kashiwa 277-8577, Japan; hfuchiga@east.ncc.go.jp (H.F.); mayasuna@east.ncc.go.jp (M.Y.); 6 Shimadzu Corporation, Kyoto 604-8511, Japan; 7 Division of Developmental Pathology, Exploratory Oncology Research & Clinical Trial Center, National Cancer Center, Kashiwa 277-8577, Japan; ssakashi@east.ncc.go.jp; 8 Division of Science and Technology for Endoscopy, Exploratory Oncology Research & Clinical Trial Center, National Cancer Center, Kashiwa 277-8577, Japan

**Keywords:** photoimmunotherapy, fluorescence imaging, real-time imaging, IR700, antibody–drug conjugate, anticancer therapy

## Abstract

**Background/Objectives**: Near-infrared photoimmunotherapy (NIR-PIT) was recently approved for the treatment of unresectable locally advanced or recurrent head and neck cancers in Japan; however, only one clinical dose has been validated in clinical trials, potentially resulting in excessive or insufficient dosing. Moreover, IRDye700X (IR700) fluorescence intensity plateaus during treatment, indicating a particular threshold for the antitumor effects. Therefore, we investigated the NIR laser dose across varying tumor sizes and irradiation methods until the antitumor effects of the fluorescence decay rate plateaued. **Methods**: Mice were subcutaneously transplanted with A431 xenografts and categorized into control, clinical dose (cylindrical irradiation at 100 J/cm², frontal irradiation at 50 J/cm²), and evaluation groups. The rate of tumor IR700 fluorescence intensity decay to reach predefined rates (−0.05%/s or −0.2%/s) until the cessation of light irradiation was calculated using a real-time fluorescence imaging system. **Results**: The evaluation group exhibited antitumor effects comparable to those of the clinical dose group at a low irradiation dose. Similar results were observed across tumor sizes and irradiation methods. **Conclusions**: In conclusion, the optimal antitumor effect of NIR-PIT is achieved when the fluorescence decay rate reaches a plateau, indicating the potential to determine the appropriate dose for PIT using a real-time fluorescence monitoring system.

## 1. Introduction

Near-infrared photoimmunotherapy (NIR-PIT) was first reported by Kobayashi et al. in 2011 as a novel method for tumor-specific cancer treatment [1]. NIR-PIT exerts its mechanism via a chemical reaction to form a ligand between a monoclonal antibody, such as cetuximab, which targets certain epidermal growth factor receptors (EGFRs), and the near-infrared phthalocyanine dye IRDye700X (IR700) with subsequent laser irradiation at 690 nm. IR700 exhibits fluorescence when exposed to NIR, which is quantified by the fluorescence imaging system [2]. IR700 exhibits a change in hydrophilicity to form aggregates in an aqueous medium owing to hydrophobic interactions when exposed to near-infrared light (NIR) at 690 nm. After intravenous injection, cetuximab conjugated with IR700 (Cet-IR700) binds to EGFRs on cancer cell membranes. NIR exposure induces physical stress, increasing transmembrane water flow and leading to rapid necrotic cell death [3]. NIR induces cell death only in cells bound to Cet-IR700; therefore, NIR-PIT is believed to be a treatment that combines tumor specificity with minimal side effects.

NIR-PIT for patients with unresectable head and neck squamous cell carcinoma was assessed in a Phase I/IIa trial (RM-1929-101) between 2015 and 2017 [4]. Following favorable outcomes, a Phase III trial (RM-1929-301) was conducted in 2019 [5]. Based on the favorable outcomes of the RM-1929-101 trial, NIR-PIT with Cet-IR700 was approved by the Pharmaceuticals and Medical Devices Agency of Japan in 2020. This treatment has been available for insured patients with unresectable locally advanced or locally recurrent head and neck cancer since January 2021 [4,6] and has been implemented across several facilities in Japan [7,8,9]. Head and neck cancer ranks as the sixth most common type of cancer, with a remarkable demand for photoimmunotherapy [10].

However, studies have reported patients requiring several NIR-PIT sessions owing to recurrence following the treatment [8,9]. The causes of local recurrence after NIR-PIT require further research because existing clinical investigations are insufficient. Notably, no study has ascertained a significant correlation between the irradiation dose and tumor cell death. This suggests that the antitumor effect is not solely determined by the irradiation dose, which aligns with previous reports indicating a lack of correlation between the irradiation dose and the antitumor effect [11]. Additionally, adverse events such as tracheostomy requirements due to local edema induced by NIR-PIT have been documented [12,13]. Consequently, it is critical to not only increase the irradiation dose but also to determine a dose that is specifically tailored to each individual case.

Our research group previously focused on determining the appropriate irradiation dose for NIR-PIT. A preceding report published by our research group on real-time fluorescence imaging during NIR-PIT revealed a correlation between the tumor growth inhibition effect of the therapy and the fluorescence decay rate of IR700 in the tumor. Specifically, we found that the irradiation dose representing the threshold for the antitumor effect of NIR-PIT appeared to be the dose at which the fluorescence decay rate reached a plateau [14]. Fluorescence decay rates can exhibit a plateauing effect, occurring as the fluorescence signal reaches a constant or near-constant level after the initial decay phase. This plateau phase can be attributed to various factors within the system under investigation. One common reason for observing a plateau is the presence of a long-lived component in the fluorescence decay, resulting in a gradual decline in the fluorescence signal over an extended period.

Based on this, we developed a system that stops NIR irradiation based on the tumor’s fluorescence decay rate using a real-time fluorescence imaging system termed LIGHTVISION. In this study, we investigated whether the NIR laser dose corresponding to the plateau in fluorescence decay rate can serve as the threshold for the antitumor effect of NIR-PIT across varying tumor sizes and irradiation methods. We hypothesized that the plateau of the fluorescence decay rate may be an indicator of the most appropriate irradiation dose. 

## 2. Results

### 2.1. Characterization of Cet-IR700 In Vitro Experiments

**Figure 1:**
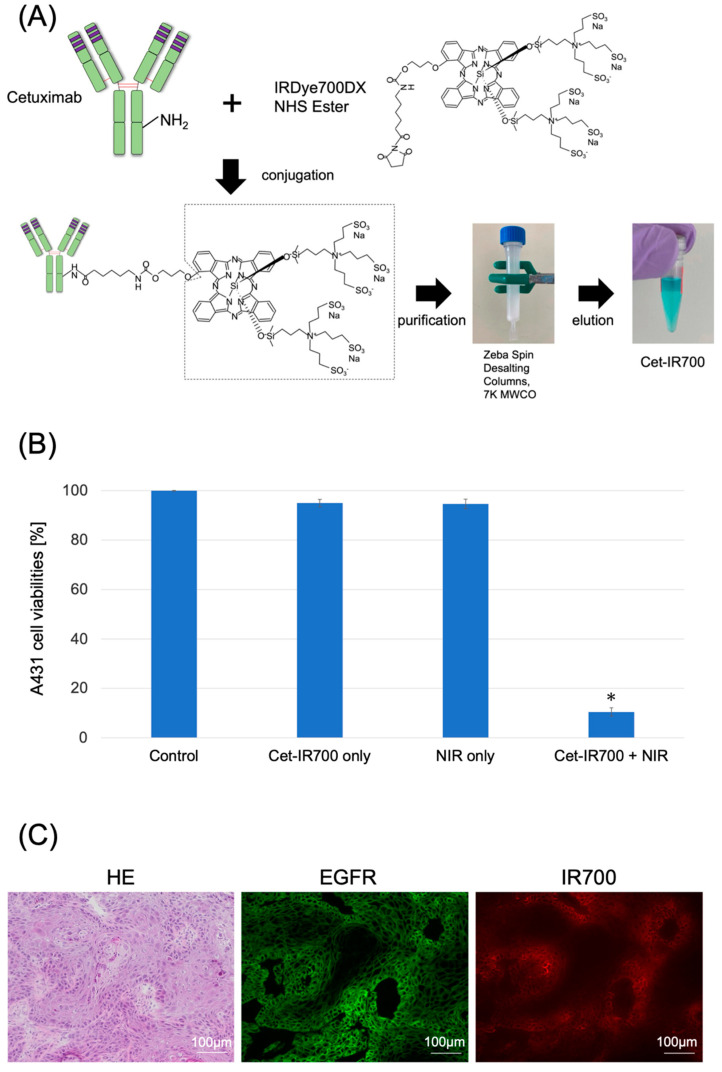
Characterization of Cet-IR700. (**A**) Production of the conjugated antibody cetuximab and IR700. Cetuximab was incubated with IRDye 700DX and eluted using Zeba Spin Desalting Columns. (**B**) Evaluation of cell death induced by photoimmunotherapy in vitro. Data are presented as means ± s.e.m. (n = 4). A431 cells were subjected to the following treatments: no treatment (control), cet-IR700 only, NIR only, or cet-IR700 + NIR. Dead cells were detected using flow cytometry. Cet-IR700 + NIR induced prominent cytotoxicity compared with the untreated control (*p* < 0.05, U test). (**C**) Histological images of A431 tumors implanted in mice using a fluorescence microscope. Mice subcutaneously implanted with A431 cells were injected with Cet-IR700 without NIR irradiation. After 48 h, cells were removed and observed under a fluorescence microscope. Anti-EGFR antibodies were bound to the plasma membrane of almost all cells, and the distribution of Cet-IR-700 fluorescence was consistent with the site of EGFR expression. Notes: * *p* < 0.05; NHS, N-Hydroxysuccinimide; MWCO, Molecular Weight Cut Off; Cet-IR700, cetuximab and IR700 conjugated antibody; NIR, near-infrared light; HE, hematoxylin and eosin stain; EGFR, epidermal growth factor receptor; s.e.m.: standard error of the mean.

Cetuximab (chimeric [mouse/human] mAb) was conjugated with IR700 following the methodology outlined in our previous study (Figure 1A and Figure S1) [11]. We performed a cytotoxicity assay with Propidium iodide (PI) staining to quantitatively examine the effects of in vitro NIR-PIT. The combination of NIR and Cet-IR700 induced prominent cytotoxicity compared to the control (*p* < 0.05) in A431 cell lines (Figure 1B).

### 2.2. Localization of Cet-IR700 in A431 Tumor In Vivo

We observed the localization of Cet-IR700 in the tumor using fluorescence microscopy. Anti-EGFR antibodies were bound to the plasma membrane of almost all cells, and the distribution of Cet-IR-700 fluorescence was consistent with the site of EGFR expression (Figure 1C).

### 2.3. Laser Light Cessation System for Photodynamic Therapy Using Real-Time Fluorescence Imaging

**Figure 2:**
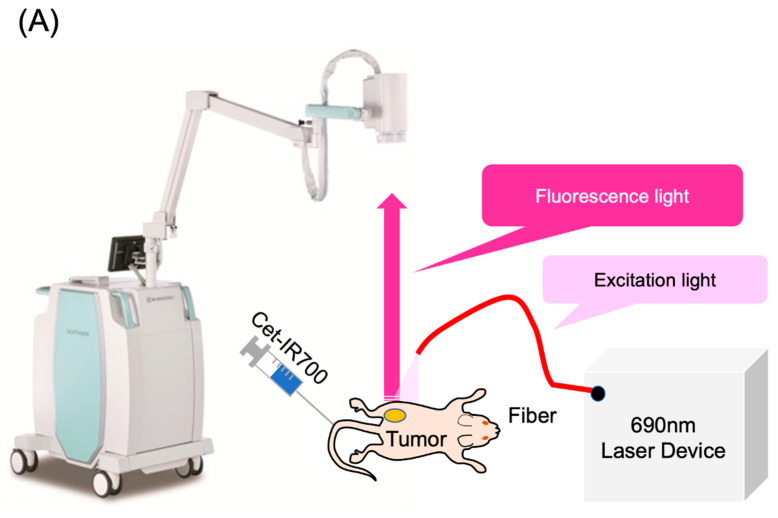

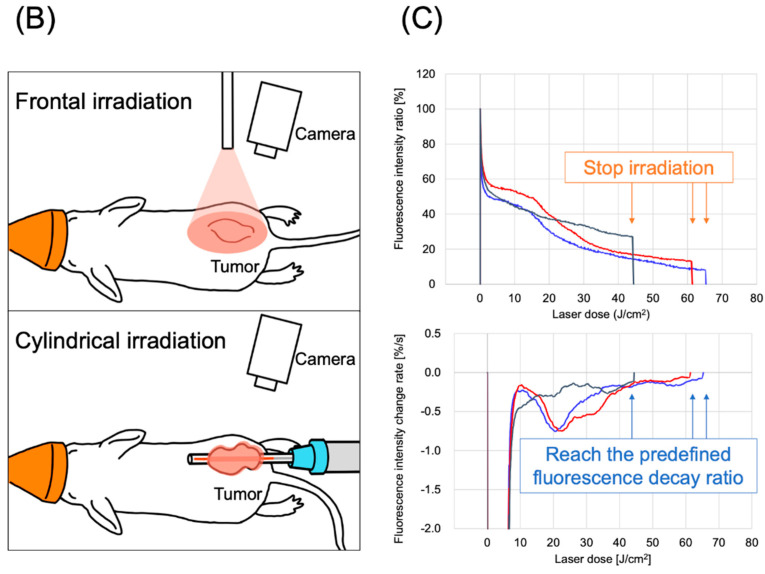
Real-time fluorescence imaging and irradiation cessation system during photoimmunotherapy in vivo. (**A**) Experimental setup for fluorescence observation during photoimmunotherapy using LIGHTVISION. (**B**) The frontal diffuser provided surface irradiation to tumors. The unit of the irradiation dose was J/cm^2^. The cylindrical diffuser provided intra-tissue irradiation of the tumor. The unit of the irradiation dose was J/cm^2^. (**C**) We measured the fluorescence intensity in real time during NIR irradiation using a fluorescence imaging system. The three lines show the fluorescence intensity ratios of three different mice with tumors. A system was established to stop laser irradiation when the predefined fluorescence decay rate was achieved. Notes: Cet-IR700, cetuximab and IR700 conjugated antibody; LIGHTVISION, real-time fluorescence imaging system developed by Shimazu Corporation; NIR, near-infrared light.

The LIGHTVISION camera system is designed to capture and image fluorescence from 825 to 850 nm, enabling detection and imaging of the location of the IR700 fluorophores. A schematic of the LIGHTVISION fluorescence imaging is shown in Figure 2A. NIR-PIT uses two irradiation methods: a frontal diffuser and a cylindrical diffuser. Both types were examined in this study, as illustrated in Figure 2B. During irradiation, we concurrently measured the fluorescence and adjusted the system to stop irradiation when the fluorescence decay rate reached a predefined value (Figure 2C). The fluorescence decay rate was calculated by averaging the decrease in fluorescence intensity rate over the last 15 s (formula described in Figure S2). The fluorescence decay curves exhibited a gradual decrease with laser irradiation and reached a plateau, mirroring trends observed in previous studies (Figure S3A–C) [14,15].

### 2.4. Antitumor Effect on Small-Sized Tumors

**Figure 3:**
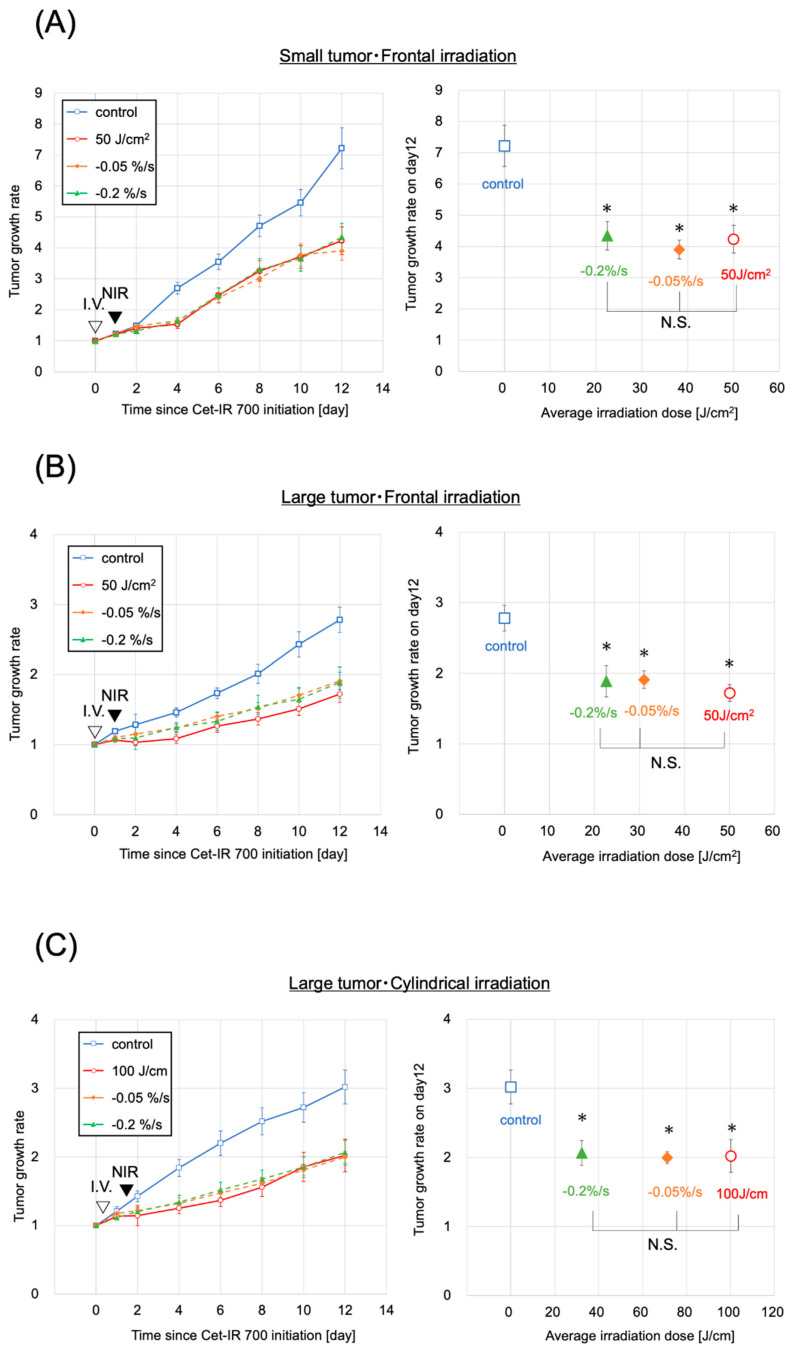
Antitumor effect by photoimmunotherapy. (**A**) Small tumor and frontal irradiation were measured. Data are means ± s.e.m. (n ≥ 11 mice in each group). (**B**) Large tumor and frontal irradiation were measured. Data are means ± s.e.m. (n ≥ 8 mice in each group). (**C**) The condition of a large tumor and frontal irradiation were measured. Data are means ± s.e.m. (n ≥ 8 mice in each group). Tumor volumes were measured from the initiation of Cet-IR700. Both the clinical dose and evaluation groups showed tumor suppression compared with the control group. Both the clinical dose group and the evaluation group showed significantly suppressed tumor growth on day 12 compared to the control group (*p* < 0.05, each group, analyzed using the Steel test). No significant difference was observed in tumor growth inhibition between the clinical dose and evaluation groups (analyzed using the Steel–Dwass test). The −0.05%/s and −0.2%/s groups demonstrated results with lower irradiation doses than the clinical doses. These results were observed under all three conditions. Notes: NIR, near-infrared light; * *p* < 0.05; N.S., not significant; Cet-IR700, cetuximab and IR700 conjugated antibody; s.e.m.: standard error of the mean.

Photoimmunotherapy was administered to mice with subcutaneously transplanted A431 cancer cell lines. The sizes of the tumors were initially controlled to range within a volume of 70–110 mm³, defined as small tumors, resembling the sizes in our previous study [14]. However, the small tumors were too diminutive to be evaluated under clinical conditions using a cylindrical diffuser. Therefore, we exclusively assessed them using a frontal diffuser. We anticipated that the antitumor effect of NIR-PIT on the small tumors would be nearly equal with frontal and cylindrical irradiation since the thickness of the small tumors was up to 1.2 mm, which was well within the achievable range of NIR. Both the evaluation and clinical dose groups demonstrated tumor growth inhibition compared to the control group (Figure 3A). The two evaluation groups and the clinical dose group exhibited significantly greater growth inhibition than the control group (*p* < 0.05, each) on day 12, the final day of the tumor size measurements in the mice. Additionally, no significant differences were observed between the evaluation and the clinical dose groups. The irradiation doses were 22 and 38 J/cm^2^ for the two evaluation groups, reaching the predefined fluorescence intensity decay rates of −0.2%/s and −0.05%/s, respectively, over the last 15 s of the fluorescence measurement until the cessation of light irradiation.

The intensity in both evaluation groups was lower than that in the clinical dose group (50 J/cm^2^) (Figure 3A). No correlations were found between the laser dose and antitumor effects (Figure S4), and no abnormal weight loss was observed in any small tumor group (Figure S5).

### 2.5. Antitumor Effect on Large-Sized Tumors

Next, we administered NIR-PIT to the larger-sized tumors, with sizes ranging from 300 to 360 mm^3^, using both frontal and cylindrical irradiation.

The conditions of large tumors and frontal irradiation were confirmed in the evaluation groups (−0.05%/s or −0.2%/s), the control group, and the clinical dose group (50 J/cm^2^), similar to the approach for small tumors (Figure 3B). Tumor growth was inhibited in the two evaluation groups and the clinical dose group, exhibiting significant tumor growth inhibition on day 12 compared to the control group (*p* < 0.05 for all). No significant difference in tumor growth inhibition effect was observed between the clinical dose and the two evaluation groups. The average irradiation doses were 31 and 23 J/cm^2^ in the evaluation groups with fluorescence decay rates of −0.05%/s and −0.2%/s, respectively, both lower than the clinical dose (Figure 3B). No correlation was observed between the laser dose and the antitumor effect (Figure S6). These findings were consistent with those observed in small tumors.

Finally, we investigated the conditions for large tumors and cylindrical irradiation. Similar to previous assessments, the evaluation groups (−0.05%/s, −0.2%/s), control group, and clinical dose group (100 J/cm^2^) were validated to assess the difference in units of irradiation dose between cylindrical and frontal conditions (Figure 3C). Tumor growth was suppressed in the two evaluation groups (−0.05%/s, −0.2%/s) and the corresponding clinical dose group, with a significant tumor growth inhibition effect (*p* < 0.05, each) observed on day 12 compared with the control group. No significant difference in tumor growth inhibition was observed between the clinical dose group and the two evaluation groups. The average irradiation doses were 71 and 32 J/cm^2^ in the groups with −0.05%/s and −0.2%/s, respectively, both lower than the clinical dose (Figure 3C). No correlation was observed between the laser dose and the antitumor effect (Figure S4). No abnormal weight loss was observed in any of the large-tumor groups (Figure S5).

### 2.6. Histological Evaluation

**Figure 4:**
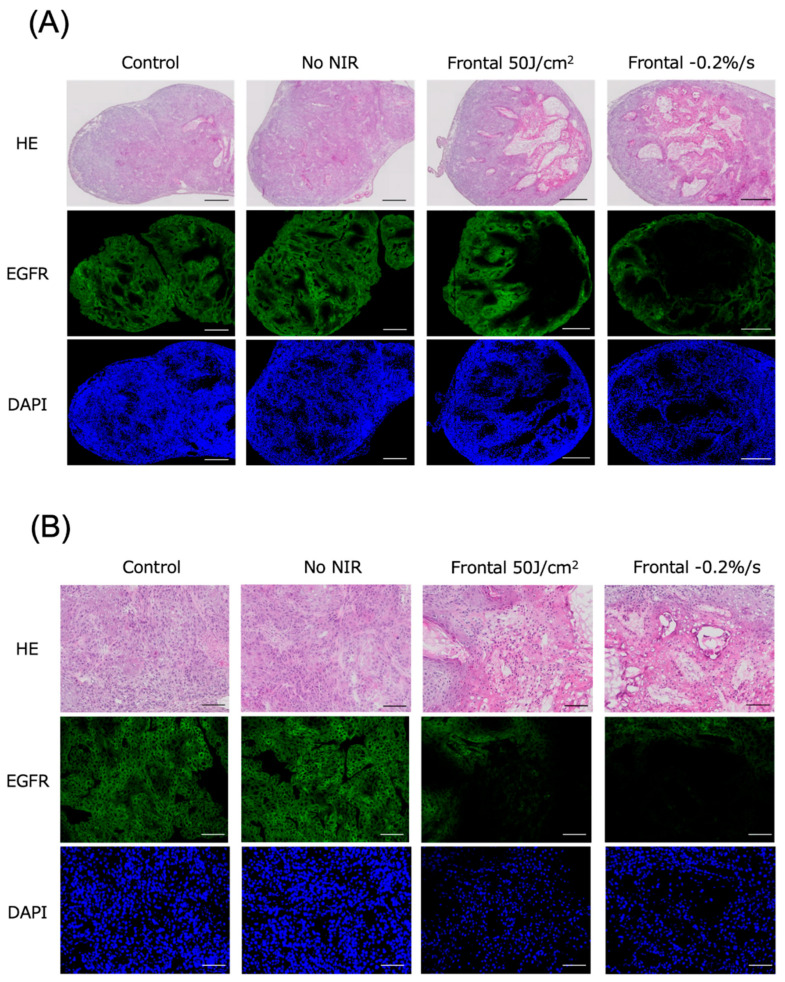
Histological findings 24 h after photoimmunotherapy using frontal irradiation. (**A**) Tumors were observed under low magnification. (**B**) Tumors were observed under high magnification. Scale bar: (**A**) 500 μm, (**B**) 100 μm. Minimal to no cell death was observed in the untreated and Cet-IR700-only groups; however, distinct cell death and reduced expression of EGFR fluorescence were noted in tumors where laser irradiation was halted at a clinical dose of −0.2%/s. Dead cells exhibited reduced EGFR expression, whereas DAPI expression persisted. Notes: HE, hematoxylin and eosin stain; EGFR, epidermal growth factor receptor; DAPI, 4′,6-diamidino-2-phenylindole.

Subcutaneous tumors in the mice were excised and histologically evaluated 24 h after NIR irradiation for photoimmunotherapy. Tumors were examined in the control, no NIR (only Cet-IR700 injected), clinical dose, and −0.2%/s groups, which had the lowest average irradiation dose among the evaluation groups. Minimal to no cell death was observed with frontal irradiation in the untreated and Cet-IR700-only tumors. However, a granular layer emerged within the tumor at the clinical dose and laser cessation at −0.2%/s, involving cytoplasmic eosinophilic changes and nuclear pycnotic changes suggestive of melt necrosis. Additionally, the nuclei in this region were identifiable by 4′,6-diamidino-2-phenylindole (DAPI) staining, but they exhibited pyknosis. Furthermore, EGFR staining did not reveal cell membranes, suggesting the degeneration and necrosis of the tumor cells (Figure 4A,B and Figure S7A). Similar pathological tendencies were observed with cylindrical irradiation as those with frontal irradiation (Figures S6A,B and S7B).

## 3. Discussion

In this study, we confirmed that the NIR laser dose at which the fluorescence decay rate reaches a plateau represents the threshold for the antitumor effect of NIR-PIT. Furthermore, these results remained consistent across different tumor sizes (small and large tumors) and irradiation methods (frontal and cylindrical irradiation).

The mechanism of NIR-PIT involves IR-700 binding to cetuximab, followed by a photoinduced ligand release reaction when exposed to NIR, inducing rapid tumor cell death [3]. An insufficient irradiation dose may reduce treatment efficacy, whereas excessive doses have been reported to increase adverse events [16,17]. Therefore, determining the appropriate irradiation dose is crucial. However, the laser dose for NIR-PIT is fixed in clinical practice, and no trials have been conducted on individually modifying the laser dose during NIR-PIT [4,6]. One reason for this is the lack of a specific method for evaluating the efficacy of NIR-PIT during NIR irradiation.

The current laser system irradiated light at 689 ± 5 nm, matching the absorption peak of IR700. However, measuring the fluorescence of IR700 at 700 nm is challenging owing to the high intensity of the 689 nm laser light. Nevertheless, although IR700 exhibited a fluorescence peak at 700 nm, it also has a tail extending towards 850 nm. Thus, indirectly measuring the IR700 fluorescence across time points was possible using a camera with sufficient sensitivity [15,18,19]. IR700 undergoes a photoinduced ligand release reaction upon NIR irradiation, leading to an irreversible loss of its fluorescence. This allowed us to quantify the amount of IR700 by measuring the fluorescence. In addition, a positive correlation was observed between the rate of tumor fluorescence and antitumor effects in an in vivo study [15]. The plateau until the maximal fluorescence decay rate is likely explained by the super-enhanced permeability and retention effect induced by NIR-PIT [15,19,20].

We revealed that the evaluation groups, defined as the cessation of irradiation when the fluorescence decay rate reached a plateau, regardless of differences in size and irradiation methods, showed no statistically significant difference in the antitumor effect compared to the clinical irradiation dose groups. As a result, both evaluation groups required less irradiation than the standard irradiation doses. These results are consistent with previous findings, suggesting that the antitumor effect remained unchanged even with irradiation doses of ≥40 J/cm^2^ for frontal and cylindrical irradiation [14,15]. Specifically, based on the findings of this study, the group that discontinued NIR irradiation at −0.2%/s appeared to achieve the most balanced combination of irradiation dose and antitumor effect. Reducing unnecessary irradiation may decrease damage to normal tissues. However, the smaller sizes of mouse tumors compared to human tumors suggest that a lower irradiation dose may have been sufficient for this study. Therefore, the irradiation dose required to reach the plateau of the fluorescence decay rate may potentially be higher for human tumors.

In this study, we pathologically confirmed that cell death occurred even at irradiation doses at which the fluorescence decay rate reached a plateau. Additionally, fluorescence microscopy revealed that although dead cells exhibited reduced EGFR expression, DAPI staining persisted. This suggests the potential of NIR-PIT to quantitatively detect cell death induced by cell membrane destruction.

In summary, our findings suggest that the plateau of the fluorescence decay rate signifies the specific threshold of laser light required in PIT, as its action remains the same regardless of tumor size variations, thereby representing an optimal threshold for phototherapy, as opposed to increasing irradiation dosage. These results imply the potential for implementing optimized photoimmunotherapy by tailoring a specific laser light irradiation dose for individual patients with varying tumor characteristics, which would be further clarified with future experiments.

Nevertheless, this study had certain limitations. First, we defined the evaluation groups to require a minimum of 20 J/cm for cylindrical irradiation and 20 J/cm^2^ for frontal irradiation based on the results indicating that cylindrical irradiation at 10 J/cm^2^ was less effective than at 40 J/cm^2^ and frontal irradiation at 10 J/cm^2^ was less effective than that at 30 J/cm^2^ [14,21]. However, the antitumor influence on the cancer in the evaluation groups plateaued at doses as low as 20 J/cm^2^ for frontal and cylindrical irradiation. Therefore, even lower irradiation doses may have been adequate. Second, this study revealed that the evaluation groups that ceased irradiation at the fluorescence decay rate plateau showed no inferior antitumor effects compared with the clinical irradiation dose groups; however, complete suppression of tumor growth could not be achieved. One possible reason for this is that this study used immunocompromised mice for in vivo experiments, resulting in limited immune support. Other studies using immunocompromised mice have shown a tendency for tumor growth after the third day of irradiation; however, considering the effect of immunity in photoimmunotherapy cell death remains a topic for future investigation [16,22]. We consider the lack of experiments involving neutralizing antibodies as one of the limitations of this study. Additionally, we are currently conducting a prospective observational study evaluating the fluorescence intensity of tumors during PIT in patients with head and neck cancer to elucidate the actual changes in fluorescence intensity during PIT in clinical settings [23].

In conclusion, we assessed tumor inhibition and fluorescence rates during photoimmunotherapy irradiation and demonstrated that an optimal antitumor effect may be achieved when the fluorescence decay rate reaches a plateau. In addition, the laser doses that reached a plateau were lower than the clinical doses. These results were consistent with different tumor sizes and irradiation methods. Based on these findings, photoimmunotherapy may be optimized by customizing the laser light irradiation dose for individual patients and tumors rather than using a fixed constant dose.

## 4. Materials and Methods

### 4.1. Cells and Cell Culture

The A431 cell line used in this study was procured from the American Type Culture Collection (Manassas, VA, USA). These cells were nurtured in Dulbecco’s Modified Eagle Medium (DMEM, FUJIFILM Wako Pure Chemical Corporation, Osaka, Japan), supplemented with 10% fetal bovine serum (FBS, Thermo Fisher Scientific, Waltham, MA, USA) and 1% penicillin-streptomycin-amphotericin B suspension (FUJIFILM Wako Pure Chemical Corporation) at 37 °C in 5% CO_2_.

### 4.2. Synthesis of IR700-Conjugated Antibodies

The antibody employed in this study was cetuximab (Merck Biopharma, Tokyo, Japan), a human/mouse chimeric monoclonal antibody of the IgG1 subclass, designed to target EGFR. The light-sensitive material used was IRDye 700DX NHS Ester (IR700; C74H96N12Na4O27S6Si3, molecular weight: 1954.22), procured from LI-COR Bioscience (Lincoln, NE, USA).

The IRDye^®^ 700DX Protein Labeling Kit (LI-COR Bioscience) was employed following a previously established procedure to attach IR700 to the antibody [24,25]. In brief, 1 mg (6.6 nmol) cetuximab was incubated with 53.2 μg (27.2 nmol) IR700. The mixture was subsequently purified using a ZebaTM Desalting Spin Column, 7K MWCO (Thermo Fisher Scientific), resulting in Cet-IR700 production. The number of fluorophore molecules bound to each antibody was measured using a spectrophotometer (NanoDrop One, Thermo Fisher Scientific) by assessing the absorption at 280 and 689 nm. An average of two IR700 molecules were found to be attached to each antibody.

### 4.3. Cytotoxicity Assay

The cytotoxic effects of NIR-PIT with Cet-IR700 were determined by flow cytometric PI staining using a previously described method [26]. A431 cells (2 × 10⁵) were seeded on 12-well plates and incubated for 24 h at 37 °C. The medium was refreshed, and 10 μg/mL of Cet-IR700 was incubated for 6 h at 37 °C. The cells were washed with phosphate-buffered saline (PBS), and a phenol red-free culture medium was added. Subsequently, the cells were irradiated with a 690 nm continuous wave laser (Shimadzu special laser module). A power density of 150 mW/cm^2^ was measured using an optical power meter (S142C; Thorlabs, Newton, NJ, USA). The laser dose was 20 J/cm^2^. The cells were collected and resuspended 1 h after irradiation. PI was added to the cell suspension (final concentration: 2 μg/mL). Stained cells were examined using a Guava easyCyte 10HT flow cytometer (Merck Millipore, Billerica, MA, USA). FlowJo software (Tree Star Inc., Ashland, OR, USA) was used for data analysis.

### 4.4. Fluorescence Microscopy

Fluorescence microscopy was performed to detect the antigen-specific localization of Cet-IR700 in the tumors. Fluorescence was assessed using a BZ-X700 fluorescence microscope (Keyence, Osaka, Japan).

### 4.5. Animal Model

Six-week-old female BALB/c nu/nu mice (Charles River Japan, Yokohama, Japan) were used in this study. The mice were subjected to anesthesia using isoflurane and inoculated with 3.5 × 106 A431 cells suspended in 100 μL of PBS on the left dorsal side. The tumor volume was calculated using the formula TV = (L × W2)/2, where L and W represent the length and width of the tumor beneath the skin, respectively, and TV is the tumor volume [27]. Tumor volume and weight were assessed every two days, and mice that reached a volume of 2000 mm^3^ or experienced weight loss exceeding 20% were humanely euthanized.

The animal experiments conducted in this study received approval from the Animal Experiment Committee of the National Cancer Center Japan. All animal experiments adhered to the guidelines for the Care and Use of Laboratory Animals established by the committee. These guidelines comply with the ethical standards mandated by law and are in accordance with Japanese regulations concerning the use of laboratory animals.

### 4.6. In Vivo Real-Time Fluorescence Imaging

We employed LIGHTVISION (Shimadzu Corporation, Kyoto, Japan) to perform fluorescence imaging during NIR-PIT at a collection wavelength of ≥820 nm. The excitation source was a 690 nm laser emitted from a Shimadzu special laser module (peak wavelength 690 ± 5 nm). In NIR-PIT therapy, the laser dosage was 400 mW/cm² for cylindrical irradiation and 150 mW/cm² for frontal irradiation. In this setup, a cylindrical diffuser (RD-ML20, Medlight, Ecublens, Switzerland) responsible for emitting light from the laser device into the tumor was placed longitudinally within the tumor to facilitate intratumor irradiation. A frontal diffuser (FD1, Medlight), which emitted light onto the tumor surface, was positioned directly above the tumor. To target small tumors (70–110 mm^3^ in volume), the distance between the diffuser tip and the tumor was adjusted to ensure that the spot size was 15 mm in diameter on the tumor surface. Similarly, to target larger tumors (300–360 mm^3^ in volume), the spot diameter was set to 20 mm.

The LIGHTVISION camera head was placed approximately 50 cm from the observation target. Mice with A431 tumors were injected with 100 μg of Cet-IR700, and NIR-PIT treatment was administered after 24 h to obtain fluorescence images and the tumor fluorescence intensity during treatment.

Before light irradiation, the region of interest (ROI) was set within the tumor area of the visible images using Shimadzu real-time fluorescence analysis software, which corresponded to the same region in the infrared fluorescence images. During light irradiation, fluorescent images were acquired at a rate of 10 frames/s, and the average pixel value in the ROI of each image was calculated in real time. The average pixel value was normalized to the average pixel value in the ROI at the start of light irradiation, and the result was defined as the fluorescence intensity ratio, expressed in % units.

The difference between the fluorescence intensity ratio at a certain time (t0) and 15 s earlier (t0 − 15) was divided by 15 s. The average difference from the most recent 15 data points was then calculated, and this average was defined as the fluorescence intensity ratio decay rate, expressed in %/s units.

The following conditions were defined as the plateau conditions, in which the fluorescence decay rate reached equilibrium: (a) −0.05%/s and an energy amount of ≥20 J/cm^2^ (defined as the −0.05%/s group) and (b) −0.2%/s and an energy amount of ≥20 J/cm^2^ (defined as the −0.2%/s group).

### 4.7. In Vivo NIR-PIT with Cet-IR700

The in vivo experiments were performed using mice implanted with A431 cells. For the small tumor model with frontal irradiation, mice with A431 tumor xenografts and a tumor volume of 70–110 mm^3^ were randomly divided into four groups (n = 11–12 mice/group), as follows: (a) no treatment (control group), (b) −0.05%/s group, (c) −0.2%/s group, and (d) clinical irradiation condition (50 J/cm^2^).

For the larger tumor models with frontal irradiation, mice with a tumor volume of 300–360 mm^3^ were selected and randomly categorized into four groups (n = 8–10 mice/group), similar to those used in the small tumor model. For the larger tumor model with cylindrical irradiation, mice with a tumor volume of 300–360 mm^3^ were selected and randomly divided into four groups (n = 8–9 mice/group), similar to those used in the small tumor model. Laser irradiation was performed under isoflurane anesthesia, and a 690 nm continuous wave laser was used at a power density of 150 mW/cm^2^ for frontal irradiation and 400 mW/cm^2^ for cylindrical irradiation. After NIR-PIT treatment, the tumor volume was measured once every two days until day 12.

The measurements of mouse tumor size and body weight were conducted by individuals different from those who evaluated the results.

### 4.8. Histological Analysis

A series of histological changes were evaluated 24 h after NIR irradiation. Mice with A431 tumors were randomly divided into four conditions, as follows: (a) no treatment (control group), (b) Cet-IR700 injected without NIR irradiation, (c) −0.2%/s group, and (d) clinical irradiation condition (frontal irradiation, 50 J/cm^2^; cylindrical irradiation, 100 J/cm^2^). The laser dosage was 400 mW/cm^2^ for cylindrical irradiation and 150 mW/cm² for frontal irradiation, similar to our previous experiments [14]. The tumors were subsequently excised and frozen at −80 °C with an optimal-cutting-temperature compound (Sakura Finetek Japan, Tokyo, Japan). Frozen tumors were sliced into 6 μm serial sections and processed for fluorescence microscopy, EGFR immunofluorescence staining, and hematoxylin and eosin (H&E) staining. H&E staining was performed according to standard procedures.

For EGFR immunostaining, the sections were fixed in 10% neutral buffered formalin (Muto Pure Chemicals, Tokyo, Japan). Non-specific sites were blocked with 4% block ACE (KAC, Hyogo, Japan) for 30 min at room temperature. Sections were incubated with rabbit anti-EGFR antibody (Abcam, Cambridge, UK) overnight at 4 °C, followed by incubation with the appropriate Alexa Fluor 488 secondary antibody (goat anti-rabbit IgG (H+L) Highly Cross-Adsorbed Secondary Antibody, Alexa Fluor™ Plus 488, Thermo Fisher Scientific) for 1 h at room temperature. The cells were counterstained with DAPI. Stained slides were mounted and imaged using a BZ-X700 fluorescence microscope, and the images were analyzed using a BZ-X analyzer (Keyence).

### 4.9. Statistical Analysis

Statistical analyses were conducted using the R Commander software [28]. The Mann–Whitney U test was used to compare the treatment effects with the no-treatment control in the in vitro experiments as each contained only four samples, necessitating the use of a non-parametric method. The Steel test was used for multiple comparisons involving the control group. The Steel–Dwass test was used for multiple comparisons among the treatment groups (all groups except the control group). Spearman’s correlation coefficient was used to assess the correlation between the NIR dose and antitumor effect. Statistical significance was set at *p* < 0.05.

## Data Availability

The data that support the findings of this study are available from the corresponding author (Tomonori Yano), upon reasonable request.

## Supplementary Materials

The following supporting information can be downloaded at: https://www.mdpi.com/article/10.3390/ph17091246/s1, Figure S1: Structure of Cet-IR700; Figure S2: Method for determining the fluorescence decay rate; Figure S3: Real-time fluorescence imaging analysis of IR700; Figure S4: Correlation between antitumor effect and irradiation dose; Figure S5: Body weight of mice after photoimmunotherapy; Figure S6: Histological findings 24 h after photoimmunotherapy using cylindrical irradiation; Figure S7: Histological observations 24 h after photoimmunotherapy—magnified view.
