# Supplementary figures and images for: Real-Time Fluorescence Monitoring System for Optimal Light Dosage in Cancer Photoimmunotherapy
Source: Pharmaceuticals (Basel). 2024 Sep 22;17(9):1246. doi: 10.3390/ph17091246 (PMC11435081; doi:10.3390/ph17091246)

**Fig. S1**

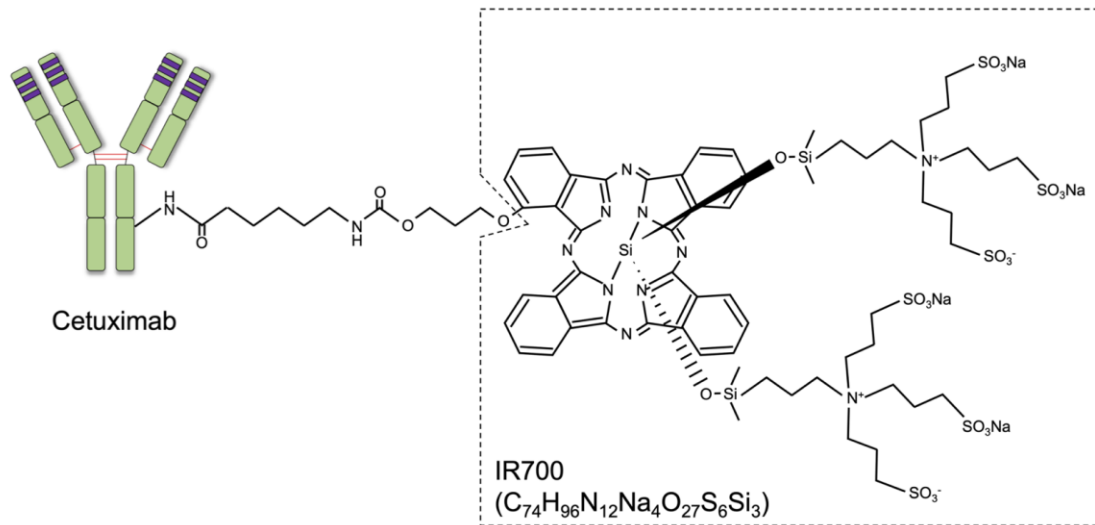

**Fig. S2**

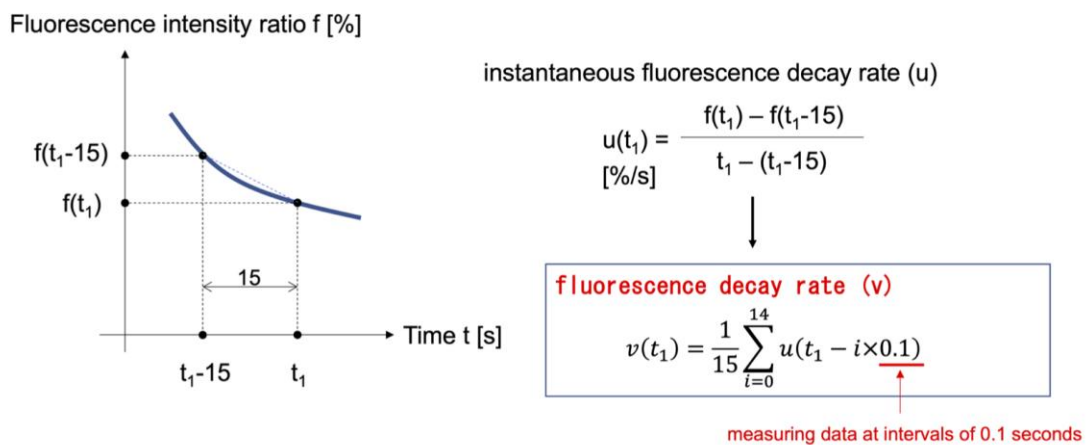

**Fig. S3**

**(A)**

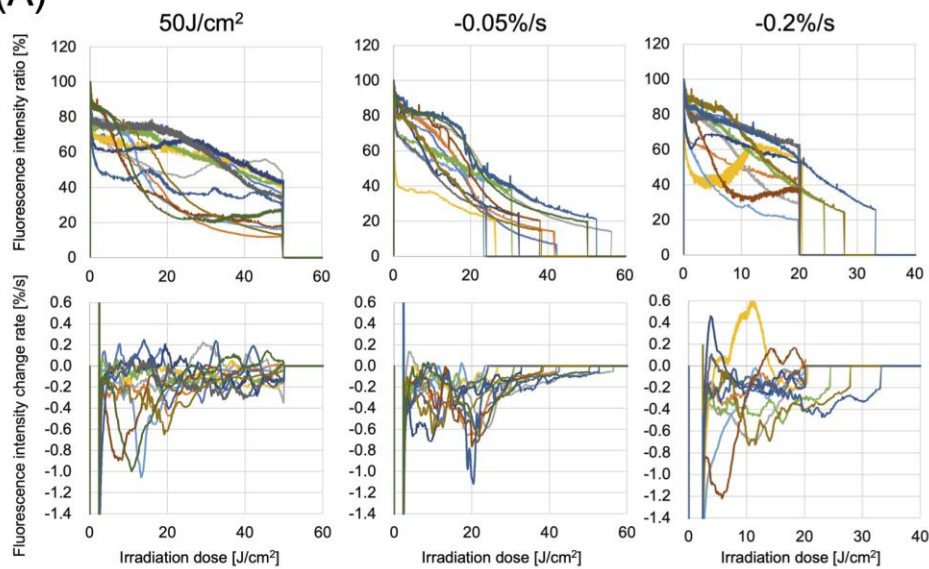

**(B)**

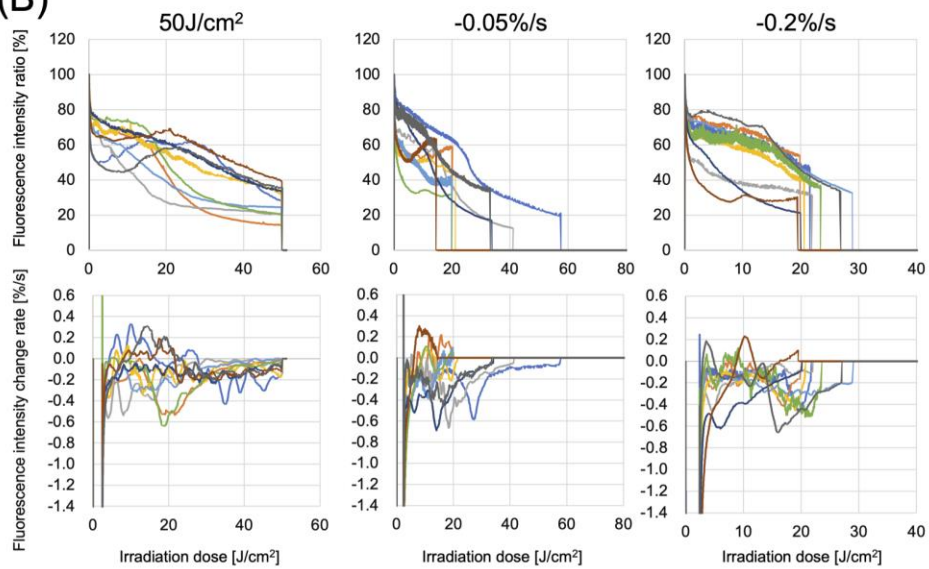

**(C)**

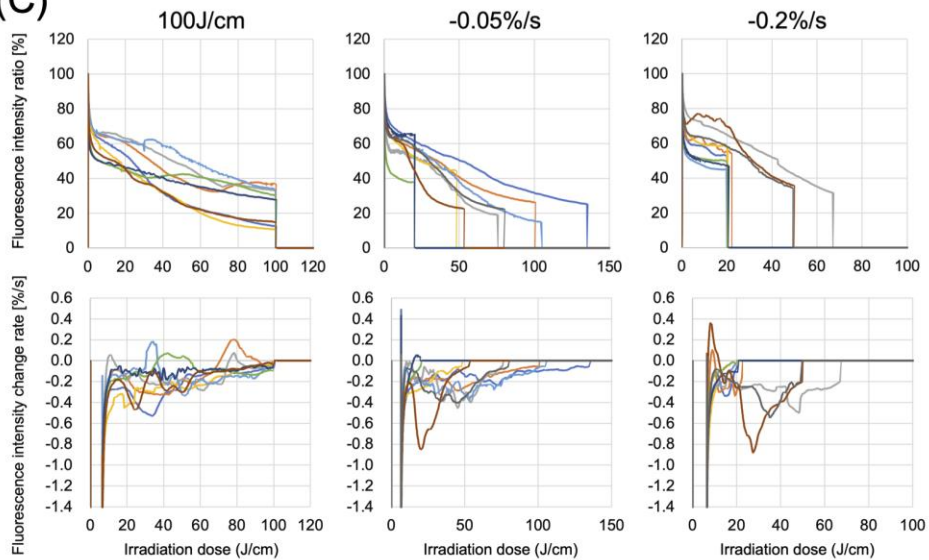

Fig. S4

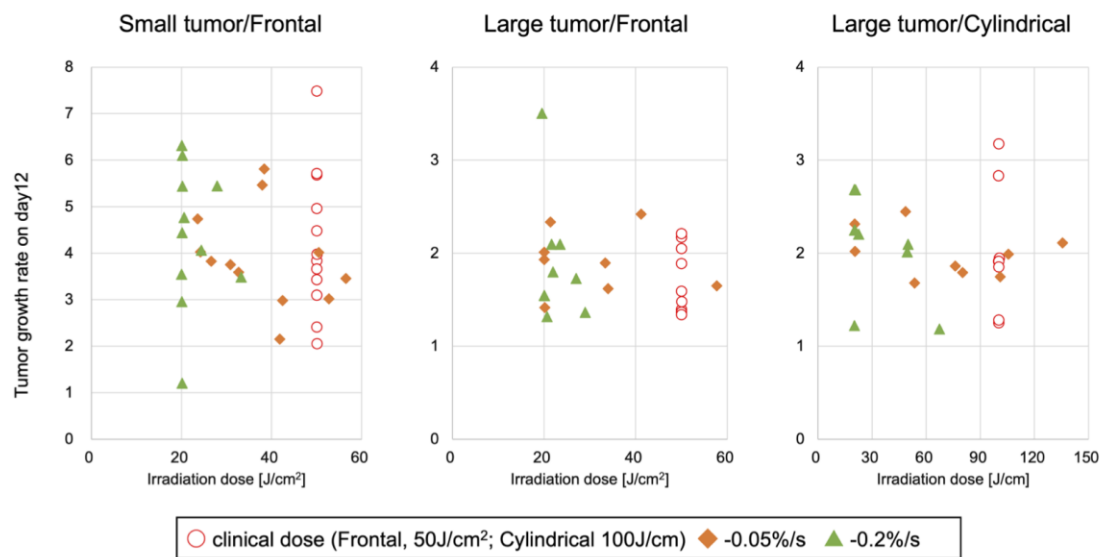

Fig. S5

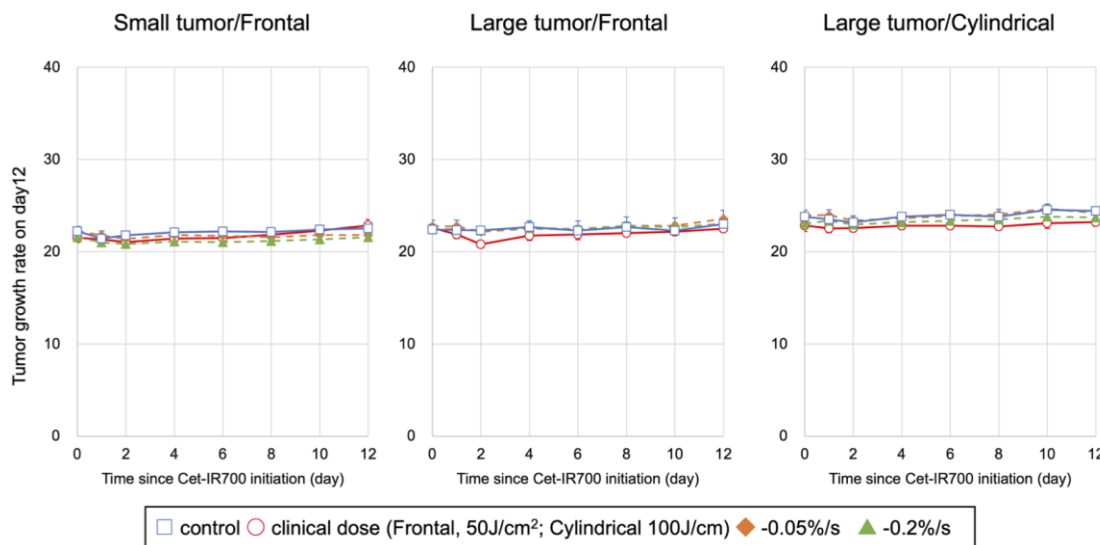

**Fig. S6**

**(A)**

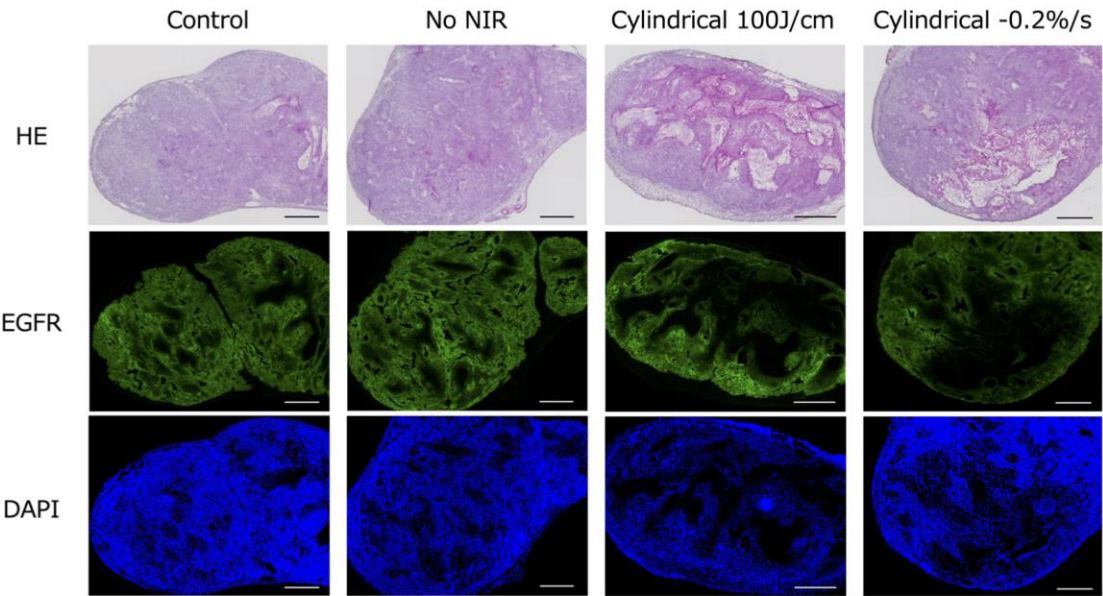

**(B)**

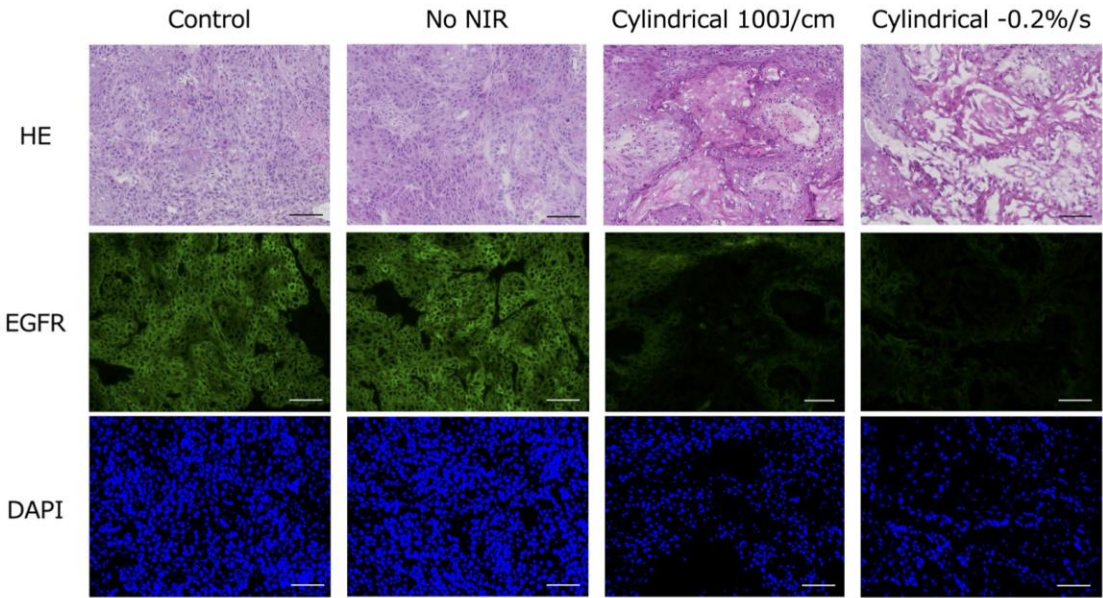

**Fig. S7**

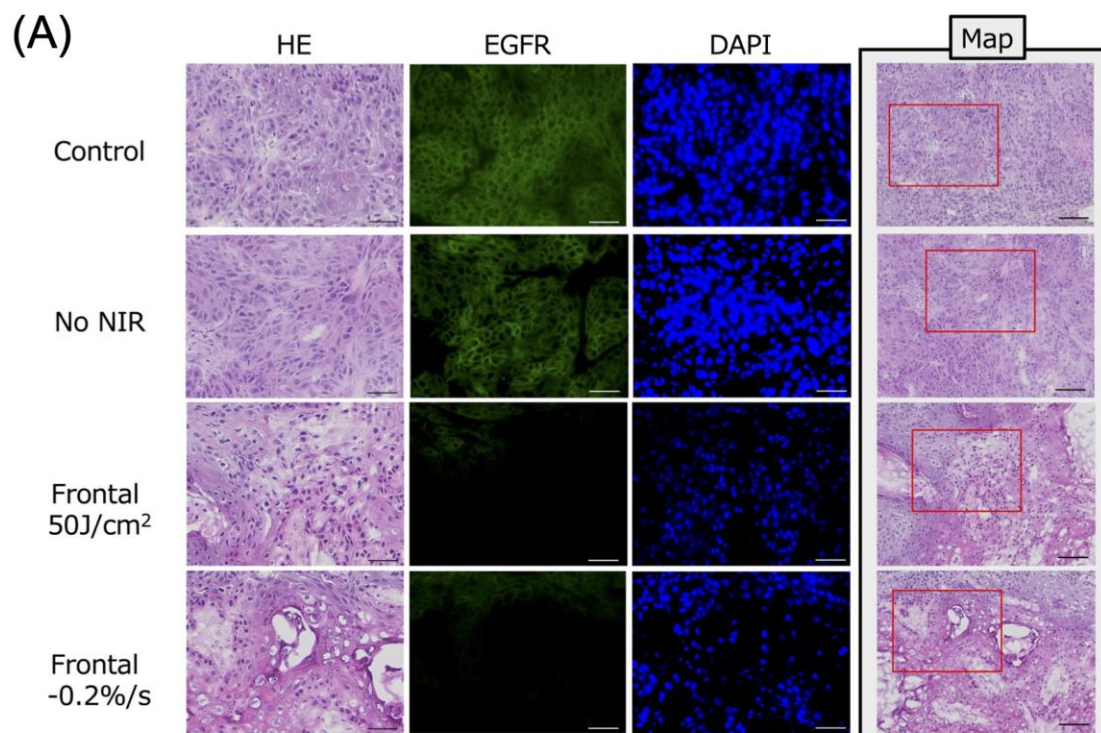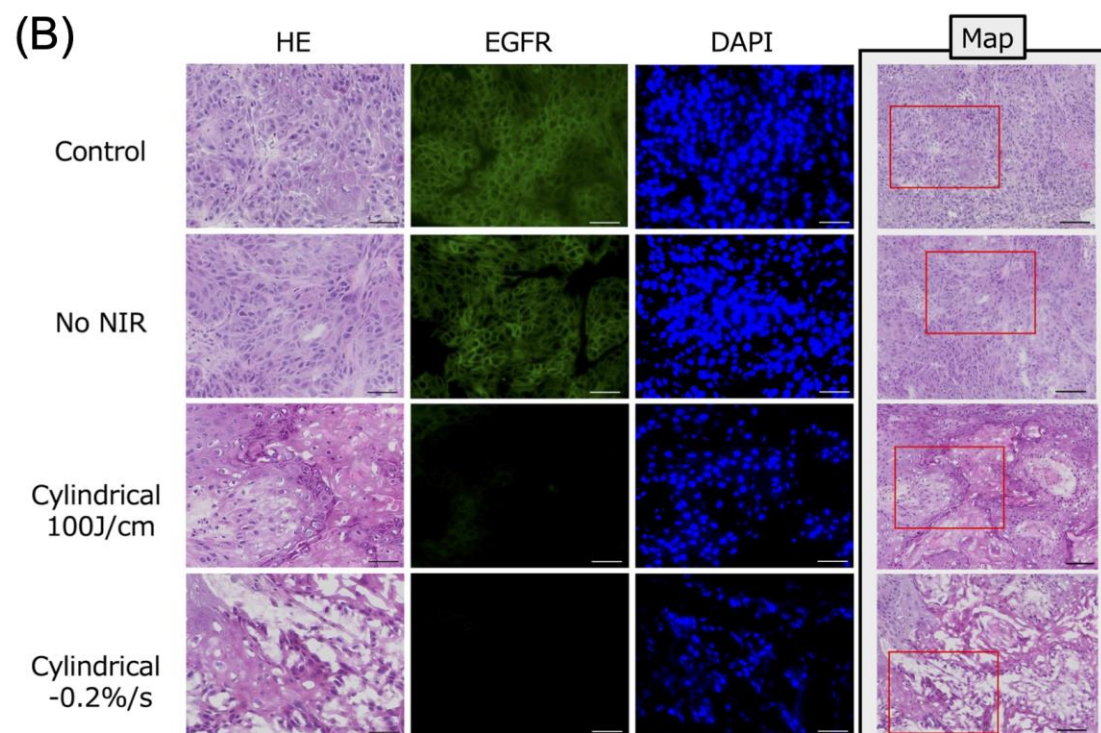

Supplement: Supplementary file 1 [file pharmaceuticals-17-01246-s001.zip › pharmaceuticals-3177831-supplementary.pdf]
